# Cranial Rosai-Dorfman disease: a case report and literature review

**DOI:** 10.3389/fonc.2024.1381958

**Published:** 2024-06-06

**Authors:** JunBo Lv, JiBo Hu, Houyun Xu, Xiping Yu

**Affiliations:** ^1^ Department of Radiology, The Fourth Affiliated Hospital of School of Medicine, and International School of Medicine, International Institutes of Medicine, Zhejiang University, Yiwu, China; ^2^ Department of Pathology, The Fourth Affiliated Hospital of School of Medicine, and International School of Medicine, International Institutes of Medicine, Zhejiang University, Yiwu, China

**Keywords:** extranodal Rosai-Dorfman disease, cranial histiocytosis, diagnostic imaging of bone lesions, temporal bone surgery, differential diagnosis in histiocytosis

## Abstract

Rosai-Dorfman Disease (RDD) is a rare, benign, idiopathic histiocytic proliferative disorder, with its occurrence in the cranial bones being particularly uncommon and prone to misdiagnosis in preoperative radiological examinations. This article reports a case of RDD in the left temporal bone. The radiological presentation of intraosseous RDD includes osteolytic bone destruction, infrequent periosteal reaction, clearly defined tumor margins, and marked uniform enhancement on contrast-enhanced scans. However, these radiological features lack specificity, highlighting the necessity of histopathological examination for a definitive diagnosis, especially for the rarer extranodal subtypes of RDD. Surgical excision of the lesion can lead to favorable therapeutic outcomes.

## Case presentation

**Figure 1:**
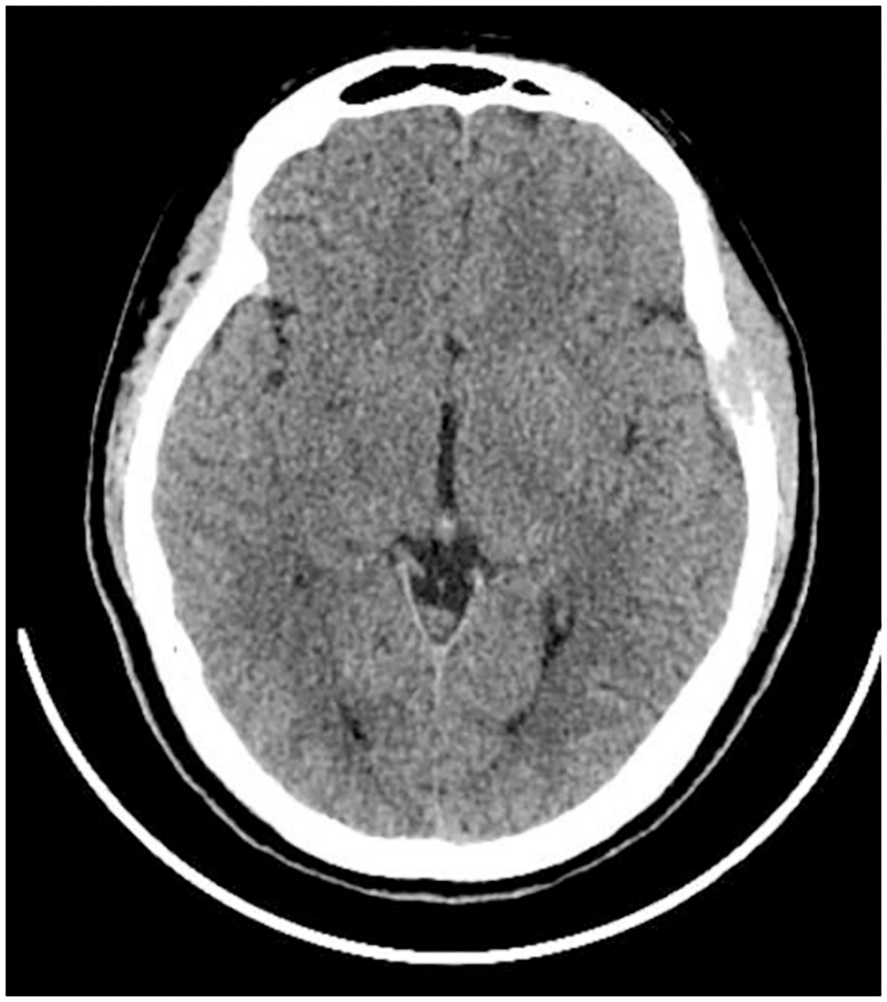
CT Scan. Bone destruction in the left temporal bone with a soft tissue density shadow, penetrating both the inner and outer tables of the cranial bone.

**Figure 2:**
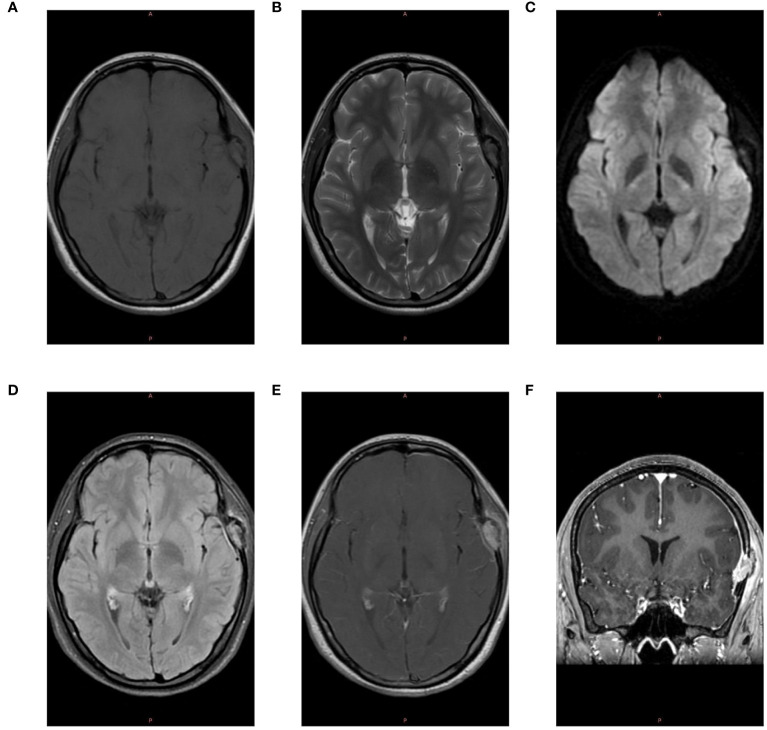
MRI Examination. **(A)** The lesion in the left temporal region exhibits isointensity on T1-weighted images. **(B)** The lesion appears iso-to-hyperintense on T2-weighted images. **(C)** The lesion shows slight hyperintensity on Diffusion-Weighted Imaging (DWI). **(D)** The lesion is slightly hyperintense on T2-FLAIR images. **(E)** Post-contrast enhancement reveals marked intensification of the lesion, with linear enhancement along the inner margin of the adjacent meninges and temporal muscle. **(F)** Coronal post-contrast scan shows marked enhancement of the lesion.

The patient, a 17-year-old female, reported noticing a mass in her left temporal region two months prior to admission, which gradually enlarged and was accompanied by localized swelling and pain. The pain subsided half a month before hospitalization. Examination revealed a firm, approximately 20mm x 20mm mass in the left temporal area, exhibiting moderate mobility, no tenderness, and no signs of erythema or ulceration of the overlying skin. Laboratory tests showed no abnormalities. CT scan revealed localized bone destruction in the left temporal bone with a soft tissue density mass breaking through the inner and outer tables of the skull, along with slight thickening of the adjacent temporal muscle (**Figure 1**). MRI scan showed localized bone destruction in the left temporal bone with a soft tissue mass, interruption of the cortical bone of the inner and outer tables of the skull, measuring approximately 22mm x 15mm. The mass appeared isointense on T1-weighted images (**Figure 2A**), iso-to-hyperintense on T2-weighted images (**Figure 2B**), slightly hyperintense on Flair (**Figure 2C**), and slightly hyperintense on DWI (**Figure 2D**). Additionally, there was slight thickening of the adjacent temporal muscle. The lesion exhibited marked enhancement on contrast-enhanced scans, with linear enhancement along the inner margin of the adjacent meninges and temporal muscle (**Figures 2E, F**).

During surgery, a fresh red-colored mass was identified in the soft tissue of the left temporal bone, penetrating the skull bone and invading the temporal muscle. The mass displayed a well-defined boundary and was richly vascularized. Using a milling cutter, the skull was milled along the edge of the tumor, revealing deep invasion of the dura mater by the tumor.

**Figure 3:**
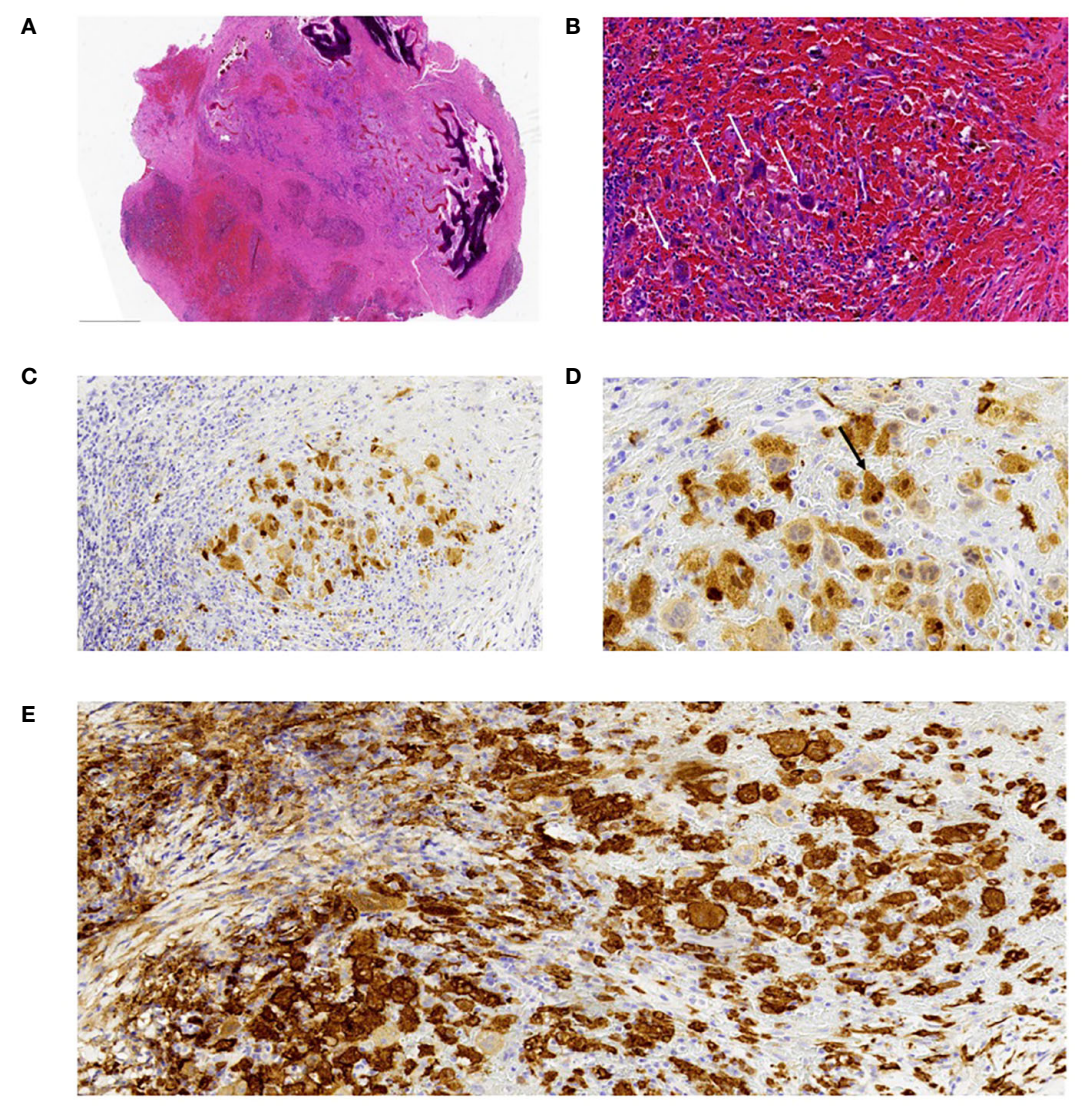
Histopathological Examination. **(A)** Under low-power magnification, the tumor is composed of areas with pale and deeply staining zones, with tumor cells showing invasive growth into and destruction of the bone tissue. Areas of stromal hemorrhage are visible. Hematoxylin and Eosin (H&E) stain, magnification x20. **(B)** At high magnification, the tumor cells are clustered in the hemorrhagic stroma, polygonal in shape, resembling multinucleated giant cells, with abundant cytoplasm and a small amount of surrounding fibrous tissue. Hematoxylin and Eosin (H&E) stain, magnification x200. **(C)** Immunohistochemistry for S100 protein shows positive expression in the cell membrane and cytoplasm. Magnification x200. **(D)** Immunohistochemistry for S100 highlights tumor cells exhibiting “emperipolesis.” Magnification x400. **(E)** Immunohistochemistry for CD163 protein shows positive expression in the cell membrane and cytoplasm. Magnification x200.

Pathology Results: Tumor cells exhibit invasive growth within bone tissue, leading to the destruction of the bone structure. The cells are spindle-shaped and polygonal, with abundant, eosinophilic cytoplasm and vacuolated chromatin (**Figures 3A, B**). Localized instances of “emperipolesis” are observed (**Figure 3D**), along with scattered fibrosis and inflammatory cell infiltration in the stroma. Immunohistochemistry: CD163 (+), CD1a (-), S-100 (+) (**Figures 3C, E**). Pathological Diagnosis: Cranial Rosai-Dorfman Disease (RDD).

## Discussion

Rosai-Dorfman Disease (RDD) is an exceedingly rare benign histiocytic proliferative disorder, initially delineated in 1969 by Rosai and Dorfman (1). It primarily affects children and adolescents, with a median onset age of around 20 years and a higher prevalence in males (2). RDD can be classified into nodal, extranodal, and mixed types, with the nodal variety being the most common (3). Clinical manifestations of RDD often include painless lymphadenopathy, fever, anemia, leukocytosis, elevated C-reactive protein, accelerated erythrocyte sedimentation rate, and hypergammaglobulinemia (4). However, some patients may present with no overt symptoms. The etiology and pathogenesis of RDD remain unclear, potentially linked to infections, genetic factors, and immune dysfunctions (5).The case occurred in the cranial bones. Rare cranio-cerebral tumors, due to limited literature reports and inadequate understanding, pose challenges for clinical practice (6).

Osteal RDD is exceedingly rare, with only a few documented case reports in domestic literature, particularly uncommon when it occurs in cranial bones. The pathological histological characteristics include: (1) In the pathological tissue, there is significant proliferation of histiocytes, which often exhibit spindle-shaped changes, arranged in a woven or whorled pattern, accompanied by a small number of lymphocytes, plasma cells, neutrophils, and eosinophils infiltrates. The interstitial fibrosis reaction is more pronounced. In contrast, nodal Rosai-Dorfman Disease commonly shows a substantial accumulation of histiocytes, lymphocytes, and plasma cells. (2) A characteristic feature of nodal RDD is the presence of phagocytic lymphocytes within histiocytes, known as the “emperipolesis”. However, insufficient number of phagocytic histiocytes in osteal RDD, leading to less pronounced “emperipolesis”. (3) Osteal RDD may exhibit aggressive growth into surrounding areas, invading and destructing bone and cartilage tissues, leading to osteolytic destruction. (4) Immunohistochemical analysis reveals CD1a-CD68+S-100+, aiding in differential diagnosis (7). The pathological manifestations in this case include histiocytic hyperplasia and significant fibrotic reactions. The “emperipolesis” are not prominent, which is consistent with previous literature reports.

The radiographic manifestations of intraosseous RDD are sparsely reported in literature. On X-rays and CT scans, it typically presents as osteolytic bone destruction with rare periosteal reactions (8). The masses are well-defined, and invasion into surrounding soft tissues is uncommon, often exhibiting a drilling-like growth pattern into areas of lesser resistance along the muscle spaces (9). On MRI, it appears as low or isointense on T1-weighted images and hyperintense on T2-weighted images, with uniform and marked enhancement post-contrast (10). In this case, the radiographic features of cranial RDD are consistent with previous reports, pathologically manifesting as a soft tissue mass originating from the left temporal bone, breaching the cortical bone and infiltrating the dura mater, in alignment with the drilling-like growth pattern described in literature. However, the involvement of the temporal muscle in this case is atypical, as intraosseous RDD generally does not invade surrounding muscles, making this case particularly distinctive.

In cases of RDD involving the cranial bones, differential diagnosis must be considered with the following conditions: (1) Langerhans Cell Histiocytosis: This primarily occurs in children and young adults, particularly males. The nuclei of Langerhans cells are oval-shaped with inconspicuous nucleoli, and large histiocytes are rare and lack the “emperipolesis” phenomenon. Immunohistochemically, these cells are positive for S-100, CD1a, and langerin. Under electron microscopy, Birbeck granules are observed. (2) ALK-positive histiocytosis: Differentiating between RDD and ALK-positive histiocytosis may pose challenges, yet immunohistochemical staining and molecular detection can discern them. ALK-positive histiocytosis exhibits positivity for ALK protein and ALK gene rearrangement, whereas RDD lacks these features. (3)Anaplastic Large Cell Lymphoma: The tumor cells exhibit a diffuse infiltrative growth pattern, with occasional presence of abundant inflammatory cells in the background and also sinusoidal infiltration observed. The tumor cells display an anaplastic morphology, characterized by large and pleomorphic nuclei, which are often horseshoe-shaped or kidney-shaped. The cytoplasm is abundant and typically clear, with multiple nucleoli visible. Immunophenotypically, the cells generally show strong positivity for CD30 in the membrane and Golgi region, while being negative for S100, serving as a distinguishing feature. (4) Metastatic Tumors of the Skull: Metastatic tumors in the skull usually occur in older patients and are often associated with identifiable primary lesions. They can present as single or multiple osteolytic destructive foci with blurred edges.

Currently, there is no consensus on the clinical management of RDD, with reported treatment strategies including surgical excision, radiation therapy, chemotherapy, and hormone therapy (2, 11). Conservative treatment may also be considered in cases where the disease progression allows. The expert consensus suggests that approximately 20% to 50% of patients with nodal/cutaneous RDD may experience spontaneous regression (12). Therefore, a strategy of observation can be adopted for patients with uncomplicated lymphadenopathy or asymptomatic cutaneous RDD, and may also be applicable to patients with asymptomatic disease in other sites. The rationale for surgical excision in this condition extends beyond common factors to more specific considerations. Firstly, the patient in this case is relatively young, aged 17, and the tumor located on the left temporal side has been gradually enlarging, causing a noticeable protrusion. Timely surgical removal can significantly improve the patient’s appearance and quality of life, avoiding the complications of extended scarring associated with delayed surgery. Secondly, given that the tumor is located in the cranial bone, delayed excision may allow for its progression into the cranial cavity, potentially leading to severe clinical symptoms and, in extreme cases, life-threatening complications.

In summary, RDD is a rare, benign, idiopathic histiocytic proliferative disorder (13), with its occurrence in the cranial bones being particularly uncommon. Radiographically, intraosseous RDD is characterized by osteolytic bone destruction, infrequent periosteal reaction, well-defined tumor margins, and uniform, marked enhancement on contrast-enhanced scans (14). However, these imaging findings lack specificity, and definitive diagnosis relies on histopathological examination, especially for the less common extranodal subtypes of RDD (2). When RDD presents in the cranial bones, careful differentiation is required from conditions such as Langerhans Cell Histiocytosis, ALK-positive histiocytosis, Anaplastic Large Cell Lymphoma, and metastatic tumors (5).

## Data availability statement

The original contributions presented in the study are included in the article/supplementary material. Further inquiries can be directed to the corresponding author.

## Ethics statement

The studies involving humans were approved by Human Research Ethics Committee of the Fourth Affiliated Hospital of Zhejiang University School of Medicine. The studies were conducted in accordance with the local legislation and institutional requirements. Written informed consent for participation in this study was provided by the participants’ legal guardians/next of kin. Written informed consent was obtained from the individual(s), and minor(s)’ legal guardian/next of kin, for the publication of any potentially identifiable images or data included in this article.

## Author contributions

JL: Writing – original draft, Investigation, Data curation. JH: Writing – review & editing, Validation, Supervision. HX: Data curation, Writing – review & editing. XY: Conceptualization, Writing – review & editing.
